# Editorial to the Special Issue “Molecular Mechanisms and Regulation in Allergy and Immune Diseases, Immunodeficiencies”

**DOI:** 10.3390/cimb46110759

**Published:** 2024-11-10

**Authors:** Kinga Lis

**Affiliations:** Department of Allergology, Clinical Immunology and Internal Diseases, Ludwik Rydygier Collegium Medicum in Bydgoszcz, Nicolaus Copernicus University in Torun, ul. K. Ujejskiego 75, 85-168 Bydgoszcz, Poland; kinga.lis@cm.umk.pl

Allergies and other immunity disorders are a current and deepening problem of the modern world, closely related to the progress of civilization. Although many mechanisms mediating allergic reactions have already been clearly explained, there are still many unclear mechanisms behind these reactions. In addition, the relationship between allergens from different sources often remains a mystery. Immunodeficiency and immune disorders are also a serious health problem in the modern world. The coexistence of various disorders of the immune system (such as allergies and immunodeficiencies) often entails diagnostic difficulties. We often face the problem that our diagnostic tools are insufficient. Addressing this issue entails the construction of new experimental laboratory methods to study the relationship between different allergens and the mechanisms of allergies and other immunologically mediated diseases [1,2,3].

This Special Issue, titled “Molecular Mechanisms and Regulation in Allergy and Immune Diseases, Immunodeficiencies”, focuses on the analysis of various mechanisms of allergic reactions, including their genetic aspects (contributions 1–4) and issues related to allergy diagnostics, including diagnostic techniques (contribution 5). An area of particular interest of this Special Issue includes atypical hypersensitivity reactions, including sensitization to surprising and rare allergens and allergic cross-reactions (contribution 5 and contribution 6) and allergic reactions associated with immune disorders and autoimmune diseases (contribution 4 and contribution 7). Significant problems and difficulties related to allergy diagnostics, especially in difficult-to-explain cases of sensitization to atypical or hidden allergens (contribution 5 and contribution 6).

The scope of this Special Issue also encompasses technical aspects of allergy diagnostics, including the latest tools and in vitro diagnostic tests currently available in routine clinical practice (contribution 8).The possibilities of using atypical and experimental diagnostic methods and tests and individual laboratory protocols for studying the mechanisms of allergy and cross-reactivity and individualized diagnostics as an element of personalized medicine were also shown (contribution 5).

Attention was also paid to new therapeutic and prophylactic methods in the treatment and prevention of allergy and hypersensitivity, including the possibilities and benefits of using probiotics [4] and immunotherapy based on allergen components [5] or natural plant ingredients [6].

The *CIMB* Editorial Office received a total of 14 manuscripts dedicated to this Special Issue. All of them were subjected to a thorough evaluation by the Editor/Academic Editors and Reviewers, who are professionals in the field of allergy and clinical or laboratory immunology. Ultimately, 11 of these submissions were published, including 8 original articles and 3 review articles.

I hope that the goals set at the beginning of this project were achieved and that the published manuscripts contain sufficient answers to the questions asked and can serve as a source of inspiration for a wide range of readers to undertake their own research and analysis on the thematic area of this Special Issue.

To all the authors whose manuscripts comprise the content of this Special Issue “Molecular Mechanisms and Regulation in Allergy and Immune Diseases, Immunodeficiencies”, I thank you for your collaboration. Without your work, this Special Issue would never have been published. I would also like to inform you that this is not the end of our adventure with this topic. Currently, a new edition of the Special Issue, “Molecular Mechanisms and Regulation in Allergy and Immune Diseases, Immunodeficiencies, 2nd Edition”, has been launched (https://www.mdpi.com/journal/cimb/special_issues/ND7228Q9TU, accessed on 15 October 2024). We invite you, valuable authors, to send in your great manuscripts (original articles and reviews) to the new edition of this Special Issue of *CIMB*. Let us co-create a successful issue together again.
